# Estimation of the Complex Dynamic Stiffness of Inflated Rubber Diaphragms in Pneumatic Springs Using Finite Element Method

**DOI:** 10.3390/s20236728

**Published:** 2020-11-25

**Authors:** Yun-Ho Shin, Jeung-Hoon Lee

**Affiliations:** 1 Department of Safety Engineering/Big Data, Chungbuk National University, Seowon-gu, Cheongju 28644, Korea; shinyh77@cbnu.ac.kr; 2 School of Mechanical Engineering, Changwon National University, Uichang-gu, Changwon 51140, Korea

**Keywords:** complex dynamic stiffness, diaphragm, pneumatic spring

## Abstract

The accurate modeling of the complex dynamic stiffness of inflated rubber diaphragms in pneumatic springs is necessary for an efficient design of vibration isolation tables for precision instruments, such as optical devices and nano-scale equipment. In addition to pressurized air, rubber diaphragms, essentially employed for the prevention of air leakage, make a significant contribution to the total complex stiffness. To reflect the effect of the dynamic stiffness of the inflated rubber diaphragm on the total complex stiffness during the initial design or design improvement stage, it is desirable to predict the complex stiffness of the inflated rubber diaphragm beforehand. In this paper, an estimation method for the complex stiffness of inflated rubber diaphragms using the commercial finite element method (e.g., ABAQUS) is proposed. The proposed method reflects their dynamic characteristics under the large static deformation by the Mooney–Rivlin and Morman’s constitutive equations. The results of comparison with experimental results indicate that the predictions obtained by the proposed method are congruent with the experimental values of the diaphragm.

## 1. Introduction

The vibration environments for precision instruments, such as optical devices and nano-scale equipment, are frequently composed of pneumatic springs, which have lower stiffness than conventional rubber or coil springs. However, as the vibration criteria for precision instruments [1,2,3] become more stringent, it is required that pneumatic springs have better isolation performance, which can be efficiently accomplished through design improvement using an accurate mathematical model for the isolation system and experimental validation. For this purpose, first, a more accurate complex stiffness model of pneumatic springs is necessary because it is difficult to estimate values and stiffness is a key element to estimate the isolation performance.

A pneumatic spring comprises a piston and a diaphragm to enclose and seal the pressurized air inside a chamber and to guide the principal axis of the pneumatic spring. The rigid piston supports the payload mass consisting of an isolation table and/or a precision instrument. The diaphragm, a rubber membrane with a relatively complicated shape, is installed for the prevention of air leakage. Therefore, the air in the pneumatic chamber eventually works together with the diaphragm as a stiffness element when the vibrations of the base or payload cause the compression/expansion of the air.

Harris et al. [4] and DeBra [5] proposed a model for pneumatic springs by describing just the stiffness characteristic of air in a chamber through the consideration of a thermodynamic relationship. However, a practical pneumatic spring exhibits not only a higher stiffness value but also higher damping characteristics than the model of the air inside the pneumatic chamber [6]. In other words, the stiffness of the air only has a limitation to represent the actual behavior of the pneumatic spring, which may be due to the effects of the diaphragm. As the diaphragm expands owing to pressurized air, it functions as another complex stiffness element in the pneumatic springs. Hence, it is very natural to include the complex stiffness of the diaphragm in the model of pneumatic springs. Research on the behavior of the diaphragm was first conducted in 1970 by Yang and Feng [7]. They proposed a method for modeling the behavior of the diaphragm, and presented a theoretical approach to mathematically solve the swollen rubber membrane using the constitutive equation derived by Mooney and Rivlin [7]. However, there was no examination of the dynamic characteristics of viscoelastic materials. Since then, research [8,9,10] has been actively conducted to determine the equivalent stiffness using constitutive equations and the geometric shapes of materials. Recently, Xu et al. [11] proposed a method of obtaining the equivalent stiffness by dividing the diaphragm into four divisions, and the effectiveness was indirectly verified using the measured transmissibility performed on the pneumatic spring system, including diaphragms and payloads. However, it was not easy to model the diaphragm accurately in an analytical manner because it is a rubber membrane with a complicated shape and it has highly nonlinear, frequency-dependent, and amplitude-dependent characteristics. In addition, this analysis has not been reviewed in terms of dynamic stiffness coefficients representing dynamic behavior properties, including the damping or dynamic behavior of viscoelastic materials. Viscoelastic materials have dependence on the dynamic behavior. Therefore, according to the behavior of interest, the appropriate constitutive equation should be used when performing analyses using the finite element method (FEM). Therefore, in studies on rubber mounts [12,13,14,15], the analysis method and input material properties are separately applied according to the behavior of interest. However, in most studies, the diaphragm applied to the pneumatic spring only measures the equivalent dynamic stiffness, including the stiffness of compressed air in the chamber, and there are relatively few studies on the examination of the dynamic deformation characteristics, such as dynamic stiffness.

Research on the overall analysis method and procedure including the behavioral characteristics of rubber using FEM is being carried out in various fields as FEM analysis [16,17,18] and pre/post-processing technologies advance. Further, hyper-elastic theory for nonlinear analysis of static large deformation using FEM analysis and design-related research based on theory have been conducted [19,20,21]. In addition, with the development of hardware and analysis tools, analysis methodologies for reviewing detailed design results and design changes have been developed and are being used in a wide variety of fields that include a lot of viscoelastic material [22,23,24]. However, the methods developed in these studies might be difficult to apply in the initial design or concept design stage because they focused on analysis methods that are more suitable for reality by increasing the accuracy of analysis modeling. In addition, their related analyses focus on the nonlinearity due to the large static deformation of viscoelastic material, thus it is difficult to provide necessary information for estimation of dynamic characteristics. In other words, there are few studies related to a methodology for extracting the characteristics to be used in the initial or conceptual design of a passive pneumatic spring [25,26] or in a performance simulation of an active pneumatic spring system [27,28] that reflects the dynamic characteristics of a diaphragm made of viscoelastic material.

In our earlier research [25,26], we indirectly estimated the complex stiffness of a diaphragm by simply subtracting the stiffness of pressurized air from the measurement of the total complex stiffness for a single chamber pneumatic spring. The estimated results obtained were highly congruent with the typical characteristics of viscoelastic materials, which mainly constitute a diaphragm. Hence, it was believed that the major portion of the estimated results was from the complex stiffness of the diaphragm. However, the results might contain effects of unknown dynamics in addition to the diaphragm. This factor has motivated us to validate the indirectly estimated results computationally using FEM. Furthermore, to facilitate the initial design or design improvement stage of the pneumatic spring, it was previously required to predict the complex stiffness of inflated rubber diaphragms. Thus, this paper presents the computation procedure for the complex stiffness of inflated rubber diaphragms using the commercial finite element (FE) method (e.g., ABAQUS) to reflect its dynamic characteristics under the large static deformation by Mooney–Rivlin and Morman’s constitutive equations. Furthermore, a comparison with the indirectly estimated results is presented.

## 2. Estimation Procedure of the Diaphragm Complex Stiffness Using FEM

### 2.1. Proposed Estimation Method

**Figure 1:**
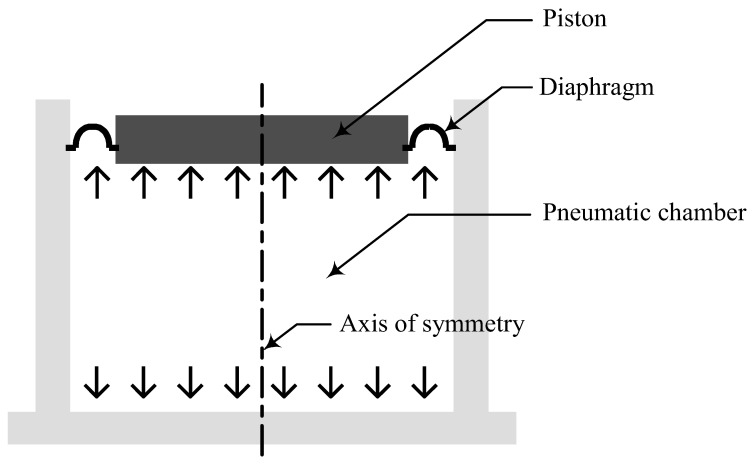
Schematic of the pneumatic spring.

The schematic diagram of a pneumatic spring is shown in Figure 1, where a piston and a diaphragm enclose pressurized air inside a chamber. The rigid piston supports the payload mass consisting of an isolation table and a precision instrument on it. The diaphragm, a rubber membrane with a complicated shape, is installed for the prevention of air leakage. The air in the pneumatic chamber eventually works together with the diaphragm as a stiffness element when the vibrations of the base or payload cause the compression/expansion of the air.

**Figure 2:**
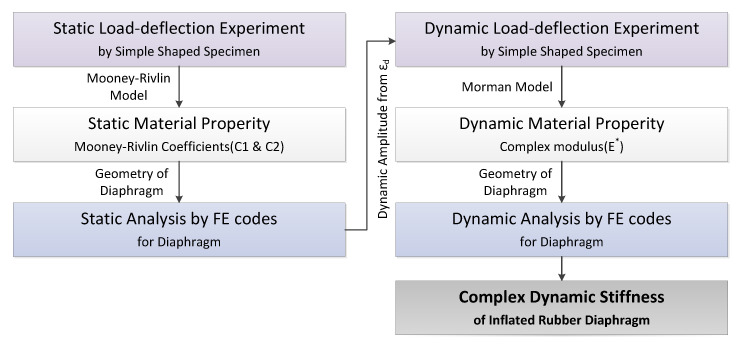
Estimation procedure of the dynamic complex stiffness of the diaphragm using finite element method.

In this study, two stages of FE analysis (i.e., nonlinear static and linear dynamic) were employed for to calculate the diaphragm complex stiffness. The objective of the nonlinear static analysis is to obtain the equilibrium configuration of the inflated rubber diaphragm after pressurization. In the linear dynamic analysis, the inflated rubber diaphragm under static equilibrium was sinusoidally excited to calculate its complex stiffness. An analysis procedure using ABAQUS, the most widely used code among commercial FE analysis codes, is proposed. The overall procedure is summarized in Figure 2. First, a simple-shape diaphragm material specimen that can easily exclude shape information is made. Then, the material property required for the Mooney–Rivlin model, a representative nonlinear constitutive equation for the static large deformation analysis, is extracted through a static load-deflection experiment, which is based on the dynamic amplitude derived through the static analysis.

### 2.2. Extraction of Material Property for the Constitutive Equation in FEM

#### 2.2.1. Extraction of the Nonlinear Static Material Property for the Mooney–Rivlin Model

**Figure 3:**
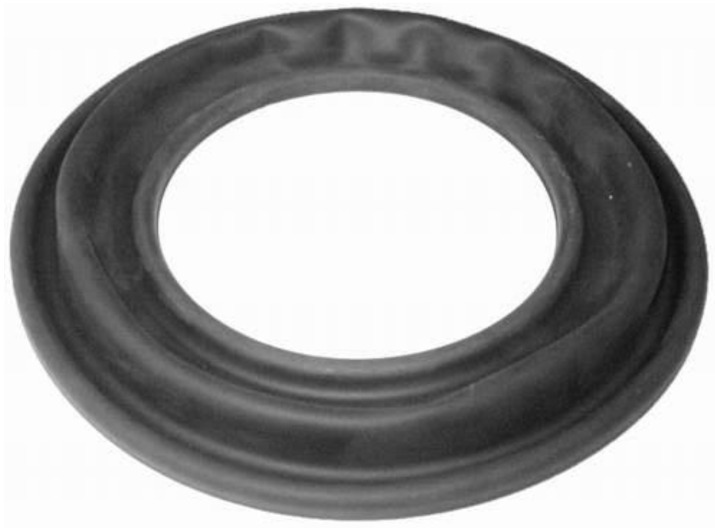
Full view of the diaphragm.

**Figure 4:**
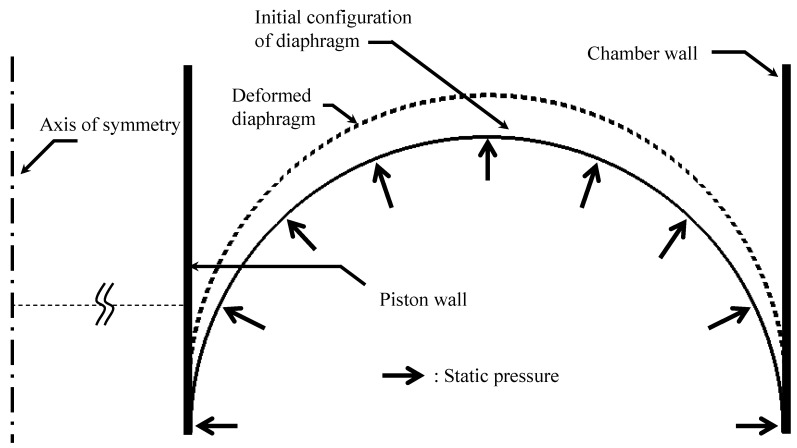
Finite element model of the diaphragm.

Because the diaphragm presented in Figure 3 experiences a large deformation (extension) during the inflation due to static pressure, a nonlinear static analysis is required. To this end, the cross-section of the diaphragm was assumed to be a semicircle. The FE model shown in Figure 4 was first constructed using one-dimensional (1D) axisymmetric elements (CAX4H [29]) based on the Mooney–Rivlin theory. The constitutive equation of the Mooney–Rivlin model [30,31,32] that can represent well the nonlinear static behavior is presented below. In a uniaxial uniform deformation, the stress σ is expressed by

(1) $${\sigma =2(C}_{1}{\lambda +C}_{2})\left(\lambda -\frac{1}{\lambda^{2}}\right),$$

where the extensional stretch *λ*( = 1 + *ε*) is related to the engineering strain *ε*. In Equation (1), the coefficients *C*_1_ and *C*_2_ are constants to be determined from the static test data.

In the case of contact condition, this work adopts the standard contact model that is readily available in ABAQUS software. In other words, it is simply performed by leaving all relevant parameters as default. The reason why the default is used and it is relatively neglected is twofold, as follows. One, the frictional characteristic between the contact pairs (the diaphragm and the piston, and chamber walls) was considered to be negligible. In other words, the walls do not seriously affect the deformation of the diaphragm, but only prevents the diaphragm from inflating in an outward direction. Another is concerned with the composition of the diaphragm. It is mainly made up of rubber material, but also reinforced by a thin fabric. The role of fabric definitely is to restrict an excessive inflation of the diaphragm, which relieves the interaction between the contact pairs.

**Figure 5:**
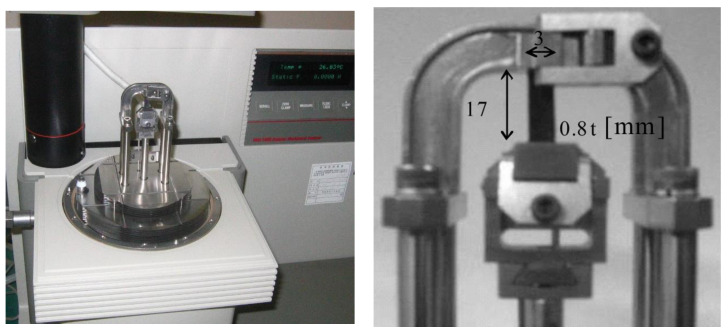
Experimental setup for the static tension test.

**Figure 6:**
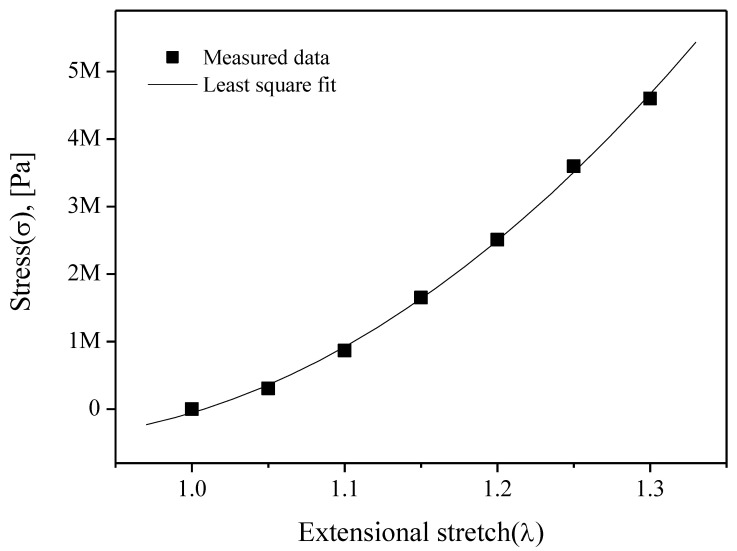
Experimental results and its least square fitting results of the static tension test, (extensional stretch (*λ*): l/l_0_).

Figure 5 shows a measurement setup for the static test, where the specimen (length: 17 mm, width: 3 mm, thickness: 0.8 mm) was installed in the material testing system (model: DMA2980, TA Instruments) driven via computer-controlled servo-electric motor actuation systems. The specimen was stretched by 5%–30%, i.e., *λ* = 1.05–1.3, and stress measurements for each stretch were made after a 20-min relaxation. The square boxes in Figure 6 represent the measurement results that determine the values of *C*_1_ and *C*_2_. The least square fit with Equation (1), when the values of *C*_1_ and *C*_2_ are 8.7 and −0.8 MPa, respectively, is shown as a solid line in Figure 6. Applying the constants to the FE model of the diaphragm can give a deformed (inflated) configuration under a static pressure, as shown by the dotted line in Figure 4.

#### 2.2.2. Extraction of the Complex Modulus for the Morman Model

One of the famous constitutive equations used to analyze the dynamic behavior of viscoelastic materials in the commercial FE analysis is the Morman model [15]. The Morman model can be simplified based on the finite linear viscoelasticity proposed by Coleman and Noll [13] and Lianis [14] and is derived to a 1D form through the following equation:

(2) $$\sigma_{d}^{*}=\left[\left(1+j\omega g^{*}(\omega ))(2C_{1}(2\lambda^{2}+\lambda^{-1})+2C_{2}(\lambda +2\lambda^{-2})\right)\right]\varepsilon_{{}_{d}}^{*},$$

where *C*_1_ and *C*_2_ were previously derived through static experiments. The relationship between $1+j\omega_{0}g^{*}(\omega_{0})$, which is a dynamic property value, and the dynamic complex modulus is summarized as follows [12]:

(3) $${1+j\omega }_{0}g^{*}{(\omega }_{0})=\frac{j\omega F[E(t)]}{E_{\infty }}=\frac{E^{*}{(\omega }_{0})}{{6(C}_{1}{+C}_{2})}=\frac{{\Delta F}^{*}{/A}_{0}\varepsilon_{d}^{*}}{{6(C}_{1}{+C}_{2})},$$

The behavior of viscoelastic materials depends on various factors, such as dynamic amplitude, static preload, and frequency. Because it is difficult to review all dependencies, this study considered only the dependence due to the frequency- and dynamic amplitude-dependent characteristics. The dynamic amplitude for a simple-shaped specimen should be defined when extracting the dynamic modulus to reflect the dependence on the dynamic amplitude.

**Figure 7:**
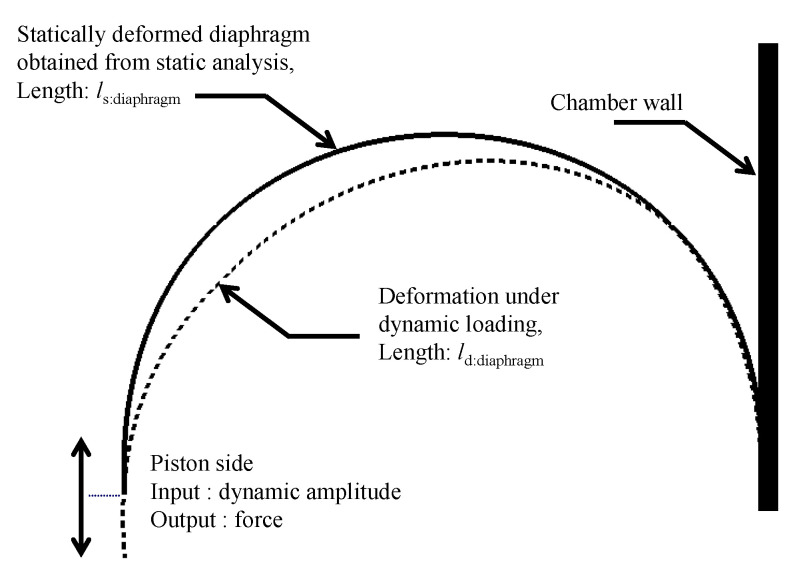
Linear dynamic analysis.

Sinusoidal displacement excitations were applied to the piston side of the statically deformed diaphragm obtained from the static analysis, while calculating the output force at that point, as depicted in Figure 7. In particular, the static pressure used in the prior analysis must be excluded to reject the transmitted force to the piston caused by pressure. In addition, the complex modulus of a typical rubber material depends on the amplitude of the dynamic strain and pre-strain [30]. Therefore, the characterization of the complex modulus subject to pre-strain *ε*_0_, which corresponds to the pressure-induced static deformation, should be conducted in the dynamic analysis stage. In this study, the following scheme was used to determine the pre-strain *ε*_0_ in the dynamic characterization:

(4) $$\varepsilon_{0}=\frac{l_{s:diaphragm}-l_{0:diaphragm}}{l_{0:diaphragm}},$$

where *l_s:diaphragm_* and *l*_0:*diaphragm*_ are the statically deformed and initial length of the diaphragm, respectively. In the same manner, the input dynamic strains *ε_d_* for the dynamic characterization can be resolved as follows:

(5) $$\varepsilon_{d}=\frac{l_{d:diaphragm}-l_{s:diaphragm}}{l_{s:diaphragm}},$$

*l_d:diaphragm_* in the above equation denotes the deformed length of the diaphragm under dynamic loading, as expressed in Figure 7. However, a bottleneck is that the dynamic deformation *l_d:diaphragm_* cannot be precisely known without complex modulus data to be measured. To obtain the approximate value of *l_d:diaphragm_*, instead, a secondary static analysis applying the dynamic displacement amplitude at the piston side was performed. Table 1 summarizes the dynamic displacement amplitude at the piston *X_p_* used in the secondary static analysis and the resulting *ε_d_* for the dynamic characterization of the specimen.

**Figure 8:**
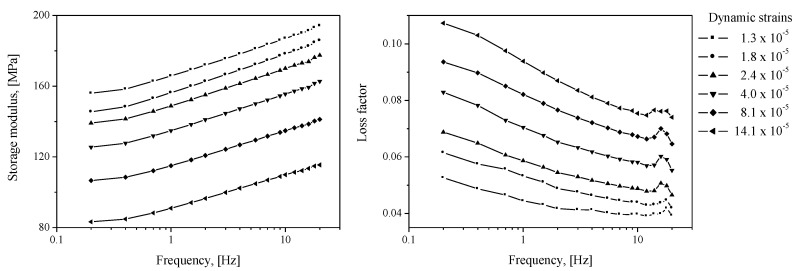
Measured complex modulus E*, *ε*_0_ = 11%; storage modulus: Re[E*], loss factor: Im[E*]/Re[E*].

By using the values of *ε_d_* superimposed on the pre-strain *ε*_0_ (11%) of the specimen, measurements of the complex modulus between 0.2 and 25 Hz were made, as presented in Figure 8.

### 2.3. Calculation of the Diaphragm Complex Stiffness

The complex modulus data were applied to the complex stiffness calculation of the inflated rubber diaphragm. For reference, different complex modulus data, which were obtained by the consideration of the static-strain distribution in the diaphragm, needed to be employed for each element of the FE model. This process may improve the quality of FE results, because the static-strain distributions of the diaphragm are not uniform. However, it is tedious and time-consuming in the state of the art of commercial FE method. Nonetheless, a systematic approach [12] can assign complex modulus data by element. However, this technique was not used in this study while assigning a single complex modulus data for all the elements.

**Figure 9:**
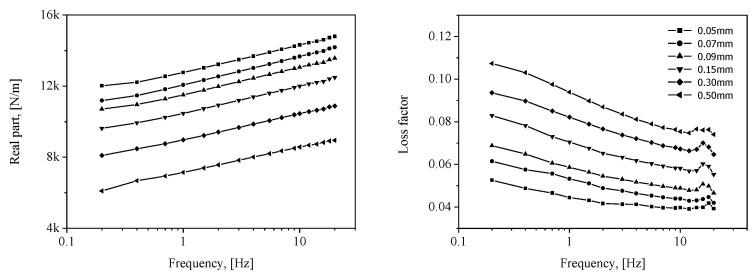
Calculated complex stiffness of the inflated rubber diaphragm k*_d_; real part: Re[k*_d_]; loss factor: Im[k*_d_]/Re[k*_d_].

The results calculated using ABAQUS, a commercial FE analysis code, are shown in Figure 9. The FE model shown in Figure 4 was also employed using one-dimensional (1D) axisymmetric elements (CAX4H [29]) based on the Mooney–Rivlin and Morman theories. The static conditions were calculated by the Mooney–Rivlin model and dynamic movement according to each frequency was analyzed by Morman’s constitutive model. Other conditions including contact condition were the same as those described in Section 2.2.1. The findings confirmed that the dependence of the dynamic stiffness on the static/dynamic deformation derived through the material property extraction experiment is well represented by the analysis results. The analysis results are compared with the experimental results presented in Section 3. In the FE analysis result of Figure 9, the phenomenon whereby the loss factor near 19 Hz partially increases is observed because the loss factor measured in Figure 8 partially increases around 19 Hz. Some increase in the measured loss factor is analyzed by the characteristics of the measuring device rather than the characteristics of the rubber material constituting the diaphragm. Many material testing devices have been observed to distort measurement results in some frequency ranges due to equipment structure, resonance, and so on. Such a phenomenon was observed in the DMA2980 device used in this study around 18 Hz, and in the INSTRON 8502 device, which measured the air spring, around 40 Hz.

## 3. Validations

### 3.1. Indirect Extraction of Diaphragm Complex Stiffness

**Figure 10:**
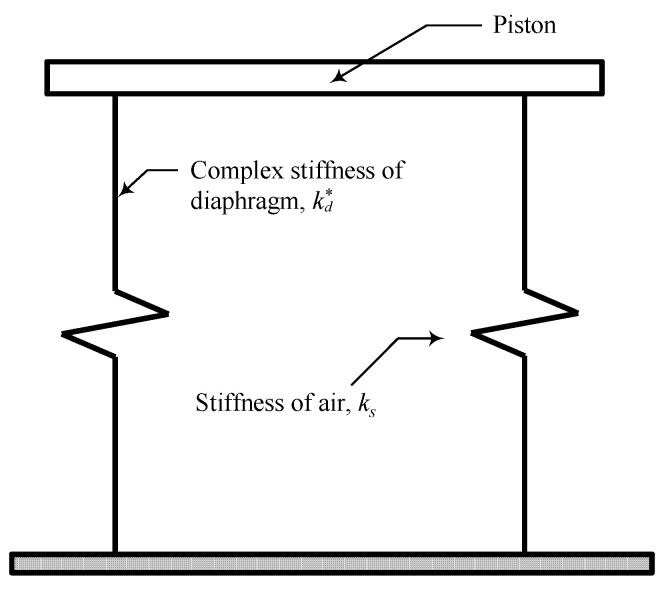
Equivalent mechanical model of the pneumatic spring.

As mentioned in the introduction, the diaphragm will deform together with the pressure change inside the chamber. That is, the measurements of the complex stiffness of the pneumatic spring contain effects of the air in the chamber and diaphragm in parallel, as shown in Figure 10. Therefore, the complex stiffness of the diaphragm can be obtained by simply subtracting the theoretical air stiffness *k_s_* from the measurements of the pneumatic spring as follows:

(6) $$k_{d}^{*}\left(X_{p},\omega \right)=k_{exp}^{*}\left(X_{p},\omega \right)-k_{s},$$

where $k_{exp}^{*}\left(X_{p},\omega \right)$ denotes the experimentally measured complex stiffness of the pneumatic spring, which may have a frequency *ω*- and dynamic amplitude *X_p_*-dependent characteristics. The stiffness of air *k_s_*, which is essential for the extraction of the complex stiffness of the diaphragm, is shown in Equation (7). Full derivation of *k_s_* requires consideration of the first law of thermodynamics and the ideal gas law in the pneumatic chamber. More details can be found in reference [25].

(7) $$k_{s}=\frac{{\kappa p}_{0}A_{p}^{2}}{V_{0}},$$

κ(=1.4) in the above equation denotes the specific heat ratio. p_0_ and *V*_0_ designate the supplied pressure and chamber volume, respectively, both of which are obtainable from direct measurements. Finally, *A_p_* represents the equivalent piston cross-sectional area under the assumption that the dynamic behavior of the piston and diaphragm can be represented by that of a single piston.

**Figure 11:**
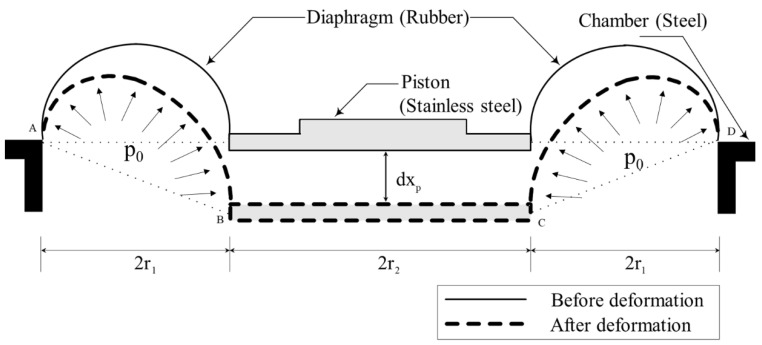
Assumption of the diaphragm movement for the calculation of equivalent piston area *A_p_*.

*A_p_* can be estimated with reference to Figure 11, which shows the deformation diagram of the diaphragm under the piston displacement of *dx_p_*. The volume variation of the top chamber *dV_t_* is approximated to be a part of a cone with cross-section ABCD, as depicted in the figure. Thus, the equivalent piston area *A_p_* is obtained by dividing *dV_t_* by *dx_p_* as follows:

(8) $$A_{p}=dV_{t}/dx_{p}=\pi \left(r_{2}^{2}+2r_{2}r_{1}+4r_{1}^{2}/3\right),$$

Using the variables described above and Equations (6)–(8), the complex stiffness of the diaphragm can be measured indirectly.

**Figure 12:**
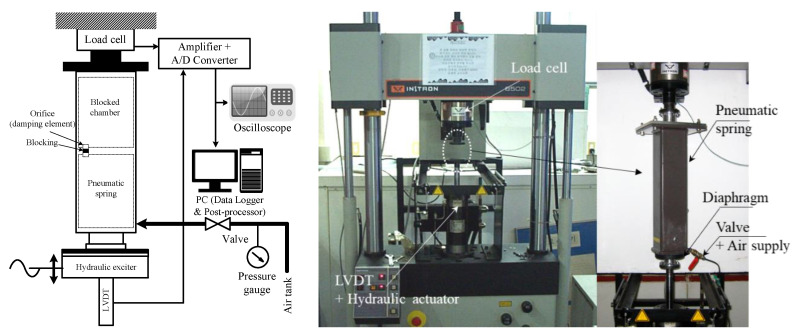
Experimental setup used for the measurements of the complex stiffness of pneumatic spring k*_exp_.

An experimental apparatus to apply the indirect measurement method explained above is shown in Figure 12. The pneumatic spring (specifications are shown in Table 2) with the applied pressure p_0_ was installed in the INSTRON dynamic material testing system (model: 8502) driven via computer-controlled servo-hydraulic actuation systems. The displacement and force signals were measured by linear variable differential transformer and load cell, respectively. The measured signals were post-processed to obtain the complex stiffness. The thick line in Figure 12 represents the pneumatic transmission line, and a pressure gauge was installed to measure the applied pressure in the chamber (i.e., the pressure at static equilibrium, p_0_). Various sinusoidal displacement excitations, all of which are the same as those in the material characterization (Table 1), were applied to the piston under a given preload corresponding to the weight of a payload mass (100 kg).

### 3.2. Comparison and Examination

**Figure 13:**
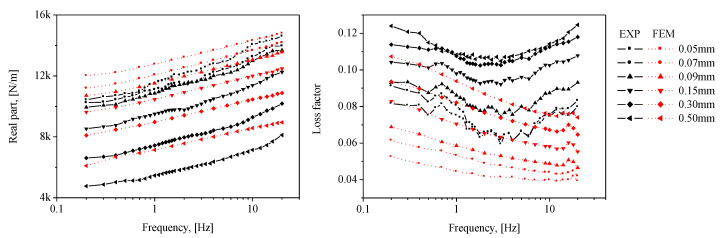
Comparison between measured and calculated complex stiffness of inflated rubber diaphragm k*_d_; real part: Re[k*_d_], loss factor: Im[k*_d_]/Re[k*_d_].

The measured complex stiffness of the inflated rubber diaphragm obtained by Equation (6) is shown as solid lines in Figure 13. The real part and loss factor are related to the stiffness and damping characteristics, respectively. First, the measured complex stiffness of the diaphragm exhibits frequency- and dynamic amplitude-dependent behaviors. More precisely, the real part representing the elastic stiffness increases with frequency and decreases with dynamic amplitude, exhibiting the behavior of a softening spring. In the case of the loss factor, it increases with the dynamic amplitude. In addition, the results for the loss factor according to the frequency show that the loss factor has a minimum point within the 1–10 Hz interval, and it might be the characteristics of viscoelastic material. However, this frequency range includes the range where the natural frequency of the air spring system, 3–5 Hz, is located when the payload is installed after designing the air spring. Therefore, it is desired that the loss factor of the diaphragm be high in this range. In this regard, the characteristics of the diaphragm extracted from the measured results will be discussed in more depth in future studies.

These observed behaviors of the complex stiffness of the inflated rubber diaphragm approximate the typical characteristics of viscoelastic materials [25,30]. From the above observations, it is reasonable to regard that the indirectly estimated complex stiffness is due to one of the diaphragms, mainly consisting of viscoelastic materials. However, the indirectly estimated results may contain the effects of unknown dynamics in addition to the diaphragm, such as nonlinearity of air due to compressibility. Hence, these experimental data need to be compared to the calculated data.

The calculation results using the FE method are signified by dotted lines in Figure 13. To examine the error between the experimental and analysis results, the mean absolute error (MAE) and root mean squared error (RMSE) defined according to Equations (9) and (10) are introduced [19].

(9) $$\mathrm{MAE}\;=\;\frac{1}{m}\sum_{i=1}^{m}\left|\frac{k_{Exp.,i}-k_{Ana.,i}}{k_{Exp.,i}}\right|$$

(10) $$\mathrm{RMSE}\;=\;\sqrt{\frac{1}{m}\sum_{i=1}^{m}{\left(\frac{k_{Exp.,i}-k_{Ana.,i}}{k_{Exp.,i}}\right)}^{2}}$$

The calculated errors by the above equations are described in Table 3. In the case of the real part, the results of the experiments and calculations exhibit qualitatively well-matching characteristics for frequency- and dynamic amplitude-dependent behaviors (the discrepancies are from 4% to 15%). Furthermore, the difference between the FE analysis and experimental results progressively decreases with a lower dynamic amplitude. The phenomenon wherein the dynamic stiffness decreases as the dynamic amplitude increases is one of the typical nonlinearities of viscoelastic material. However, in this study, Morman’s linearized constitutive equation for dynamic deformation analysis is used. As the dynamic deformation increases, the linear assumption according to dynamic amplitude dependency deviated, and it is believed that the linear dynamic analysis did not fully reflect that part even though the complex elastic modulus according to the dynamic amplitude were applied. In addition, the loss factor shows a maximum discrepancy of 40%. Practically, it is extremely difficult to obtain a good prediction quality for the loss factor of viscoelastic materials, which is also applicable in our case. In addition, based on a simple analysis of the pneumatic spring, the stiffness of the diaphragm rather than the loss factor diminishes the improvement of the vibration isolation performance of the pneumatic spring. That is, more focus on the real part of the complex stiffness is needed in evaluating the quality of calculations with FE method. By referring again to the results of the real part in Figure 13, the calculation method proposed in this study can be validated. Furthermore, a major portion of the indirectly measured results can be attributed to the complex stiffness of the diaphragm.

## 4. Conclusions

This paper discussed how to compute the complex stiffness of an inflated rubber diaphragm using commercial FE method, in which two stages of FE analysis (i.e., nonlinear static and linear dynamic) were employed. The calculated results were compared with the indirectly measured results obtained through the subtraction of the stiffness of the air in the chamber from the measured complex stiffness of the pneumatic spring. The real part of the complex stiffness, which is of primary importance in the improvement of the vibration isolation performance in the pneumatic spring, was matched well (the discrepancies ranged from 4% to 15%), and the frequency and dynamic amplitude dependency was well described through the proposed method. Thus, the calculation method proposed in this paper can be reasonably used for the calculation and prediction of the diaphragm complex stiffness, and it can be employed in the early design stage of pneumatic springs.

## Figures and Tables

**Table 1:** Dynamic amplitude at the piston *X_p_* and corresponding dynamic strain *ε_d_*.

*X_p_* [mm]	0.05	0.07	0.09	0.15	0.30	0.50
*ε_d_*	1.3 × 10^−5^	1.8 × 10^−5^	2.4 × 10^−5^	4.0 × 10^−5^	8.1 × 10^−5^	14.1 × 10^−5^

**Table 2:** Design specifications of the employed pneumatic spring for experiments.

Symbol	Name	Value	
κ	Specific heat ratio of air	1.4	
p_0_	Supplied pressure	4.93 × 10^5^	[Pa]
*V*_0_	Chamber volume	8.1 × 10^−4^	[m^3^]
*A_p_*	Effective piston area	5.3 × 10^−3^	[m^2^]
*k_s_(=*κp_0_*A^2^_p_/V*_0_*)*	Stiffness of air	23	[kN/m]

**Table 3:** Comparison between measured and calculated complex stiffness of inflated rubber diaphragm by mean absolute error (MAE) and root mean squared error (RMSE).

Dynamic Amplitude		0.05 mm	0.07 mm	0.09 mm	0.15 mm	0.3 mm	0.5 mm	Average
**Real Part**	MAE	8.4%	7.2%	4.4%	9.0%	17.8%	26.7%	12.3%
	RMSE	9.3%	7.7%	4.8%	9.4%	18.3%	27.3%	14.9%
**Loss Factor**	MAE	39.5%	32.7%	34.1%	32.8%	28.7%	21.3%	31.5%
	RMSE	39.8%	33.4%	34.7%	33.6%	29.7%	23.0%	32.8%
